# An Interpretable Model-Based Prediction of Severity and Crucial Factors in Patients with COVID-19

**DOI:** 10.1155/2021/8840835

**Published:** 2021-03-01

**Authors:** Bowen Zheng, Yong Cai, Fengxia Zeng, Min Lin, Jun Zheng, Weiguo Chen, Genggeng Qin, Yi Guo

**Affiliations:** ^1^Department of Radiology, Nanfang Hospital, Southern Medical University, Guangzhou, Guangdong 510515, China; ^2^Department of CT, Maoming People's Hospital, Maoming, Guangdong 525000, China; ^3^Department of Radiology, Honghu People's Hospital, Honghu, Hubei 433220, China; ^4^Department of Medical Services Section, Nanfang Hospital, Southern Medical University, Guangzhou, Guangdong 510515, China

## Abstract

This study established an interpretable machine learning model to predict the severity of coronavirus disease 2019 (COVID-19) and output the most crucial deterioration factors. Clinical information, laboratory tests, and chest computed tomography (CT) scans at admission were collected. Two experienced radiologists reviewed the scans for the patterns, distribution, and CT scores of lung abnormalities. Six machine learning models were established to predict the severity of COVID-19. After parameter tuning and performance comparison, the optimal model was explained using Shapley Additive explanations to output the crucial factors. This study enrolled and classified 198 patients into mild (*n* = 162; 46.93 ± 14.49 years old) and severe (*n* = 36; 60.97 ± 15.91 years old) groups. The severe group had a higher temperature (37.42 ± 0.99°C vs. 36.75 ± 0.66°C), CT score at admission, neutrophil count, and neutrophil-to-lymphocyte ratio than the mild group. The XGBoost model ranked first among all models, with an AUC, sensitivity, and specificity of 0.924, 90.91%, and 97.96%, respectively. The early stage of chest CT, total CT score of the percentage of lung involvement, and age were the top three contributors to the prediction of the deterioration of XGBoost. A higher total score on chest CT had a more significant impact on the prediction. In conclusion, the XGBoost model to predict the severity of COVID-19 achieved excellent performance and output the essential factors in the deterioration process, which may help with early clinical intervention, improve prognosis, and reduce mortality.

## 1. Introduction

Coronavirus disease 2019 (COVID-19), pneumonia caused by severe acute respiratory syndrome coronavirus 2 (SARS-CoV-2), is a highly infectious respiratory disease with a variable incubation period ranging from 1 to 14 days, and people are generally vulnerable to the virus.

Reverse transcription-polymerase chain reaction (RT-PCR) for SARS-CoV-2 is the standard for diagnosing COVID-19. However, RT-PCR takes 1–2 days to complete and may report false-negative results. Some areas even faced a shortage of RT-PCR testing kits [1, 2]. Under these circumstances, chest computed tomography (CT) played a vital role in detecting and assessing patients with COVID-19, especially in detecting patients with COVID-19 in the early stage [3].

According to clinical presentation, patients with COVID-19 were classified into four categories: mild type, moderate type, severe type, and critical type [4]. Most patients were classified as the mild type and moderate type with mild symptoms, whereas a small group of patients may experience acute respiratory distress syndrome (ARDS), septic shock, coagulation dysfunction, and multiple organ failure. These patients required ventilators and extracorporeal membrane oxygenation during an expensive treatment and had a high death rate [5]. Previous researchers showed that up to 5.0% of the patients were admitted to the intensive care unit (ICU), 2.3% of the patients needed invasive mechanical ventilation, and 1.4% of patients died eventually [6]. It is unclear why some patients develop into severe or critical cases, while others only get mild or no symptoms. The crucial factors in the deterioration process remain unknown.

Early identification of severity and crucial factors are of great value, prompting early clinical intervention and preventing deterioration of patients' condition. However, it is hard for the doctor to identify those patients under the human limitation on information processing. Hence, artificial intelligence has been widely applied in the medical domain, enabling radiologists to make full use of data, including imaging information, and explore the images' biological nature. Since the initial outbreak, attempts have been made to detect COVID-19 using chest CT.

In this study, we established a machine learning model, combining clinical information, laboratory tests, and chest CT features for early prediction of the severity and crucial factors of patients with COVID-19. Our model may help identify patients who require early clinical intervention to improve prognosis and reduce mortality.

## 2. Materials and Methods

### 2.1. Study Participants

This retrospective study evaluated de-identified data and involved no potential risk to the patients. Therefore, the institutional review board waived the requirement of obtaining written informed consent. This study included patients with COVID-19, as confirmed by RT-PCR, admitted to the People's Hospital of Honghu and Honghu Xiaotangshan Hospital from January 1 to March 27, 2020. The inclusion criteria were as follows: (a) a positive RT-PCR result for SARS-CoV-2 infection, (b) patients who underwent a chest CT scan and laboratory tests at admission in the two hospitals mentioned above, and (c) no other viral infection or serious complication. The exclusion criteria were as follows: (a) patients who underwent a chest CT scan and laboratory tests in other hospitals and (b) patients whose chest CT images showed no lesion in the lungs.

Patients' triage, sex, age, symptoms, pre-existing diseases, the temperature at admission, and laboratory tests, such as white blood cell (WBC), neutrophil, and lymphocyte counts, were collected. Patients with COVID-19 were classified into four categories [4]: (1) The mild type includes those who have mild clinical symptoms and no pneumonia manifestations found in imaging. (2) The moderate type includes the patients who have symptoms such as fever and respiratory tract symptoms with pneumonia manifestations seen on imaging. (3) The severe type fulfilled the following criteria: respiratory frequency ≥ 30/minute, blood oxygen saturation ≤ 93%, arterial partial pressure of oxygen (PaO_2_)/oxygen concentration (FiO_2_) ratio < 300, and lung infiltrates > 50% within 24–48 hours. (4) The critical type meets any of the following criteria: occurrence of respiratory failure requiring mechanical ventilation and the presence of shock and other organ failures that require monitoring and treatment in the ICU.

**Figure 1:**
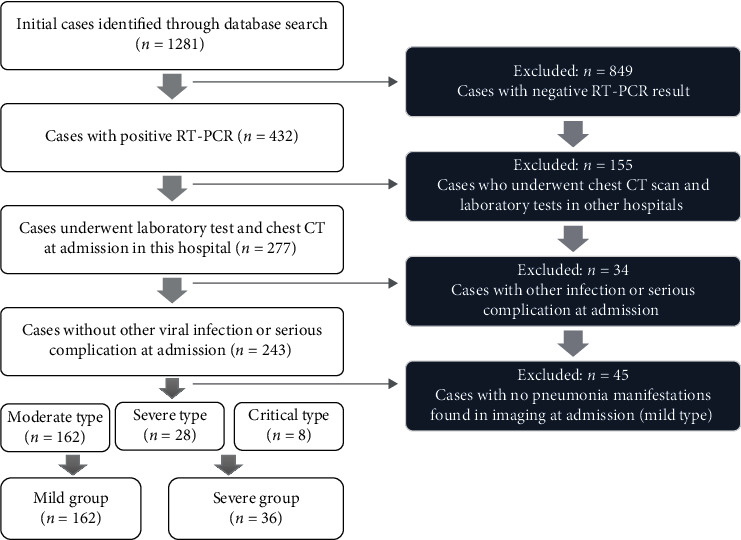
Flow diagram of patient enrollment.

In this study, all patients were classified into four clinical types according to the criteria mentioned above during treatment. The mild type was excluded because of no pneumonia manifestations found in imaging. The moderate type was classified into the mild group. Concerning the rareness of the critical type, the severe type and critical type were classified into the severe group in this study (Figure 1).

### 2.2. Imaging Techniques

Chest CT scanning (Go Now, Siemens Healthcare, Germany; GE optima 680, GE Healthcare, USA) was performed at the end of full inspiration in the supine position. The images were acquired and reconstructed with 80–130 kV tube voltage and automatic tube current modulation (up to 400 mA). The slice thicknesses were 0.6 mm (GE optima CT680) and 1.5 mm (Go Now), respectively. The lung window setting was at a window level of -600 Hounsfield units (HU) and a window width of 1500 HU. The scanning range was from the apex to the lung base.

### 2.3. Image Interpretation

All chest CT images were reviewed by two radiologists with over five years of clinical experience in the respiratory system independently. Any disagreement was resolved by discussion and consensus. The following aspects were reviewed for each patient: (1) stage (early stage, progress stage, or restoration stage); (2) distribution (subpleural, scatter, or diffuse) and shape (nodular, patchy, or large patchy); (3) number of lung lobes involved; (4) presence of ground-glass opacity (GGO); (5) presence of consolidation, fibrotic lesions, reticular shadow, crazy paving pattern, air bronchogram, pleural effusion, pleural thickening, and mediastinal lymphadenopathy (axil diameter > 10 mm); and (6) CT scores of the percentage of lung involved [7, 8]. Each lobe was evaluated for the percentage involved on a scale of 0–4 (0: 0% involvement, 1: <25% involvement, 2: 25%–50% involvement, 3: 50%–75% involvement, and 4: ≥75% involvement). The total score on the chest CT was the summation of all five lobes. The maximum possible score was 20.

### 2.4. Statistical Analysis

Statistical analyses were performed using SPSS (version 26.0). Continuous variables are expressed as means and standard deviations and compared by an independent-sample *t*-test; categorical variables are expressed as counts and frequencies (%) and compared using Fisher's exact test between the mild and severe groups. Statistical significance was set at *p* < 0.05. The area under the curve (AUC) of different models was compared by the DeLong test using MedCalc (version 19.4.1).

### 2.5. Interpretable Machine Learning Model Building

**Figure 2:**
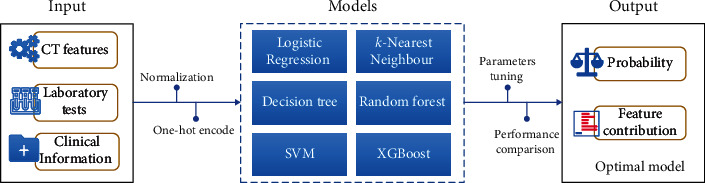
Illustration of the modeling framework.

A dataset was built, including clinical information, laboratory tests, and chest CT features, from 198 patients with COVID-19, as confirmed by RT-PCR. The machine learning model was established using Python 3.7. We randomly split the dataset into a 70% training and validation set and a 30% test set. All quantitative features were normalized to the range of 0 to 1. The categorical features were transformed into a one-hot numerical array. Six machine learning models, including logistic regression (LR), *k*-nearest neighbor (KNN), decision tree (DT), random forest (RF), support vector machine (SVM), and eXtreme gradient boosting (XGBoost), were built based on the features after preprocessing. After parameter tuning, the model's performance was assessed using the AUC. The receiver operating characteristic (ROC) curve of each model was further evaluated using DeLong's test on MedCalc (Figure 2).

Based on Shapley values from coalitional game theory, Shapley Additive explanations (SHAP) were used to explain the model [9, 10]. The SHAP explains the model prediction by computing each feature's contribution individually or jointly to the prediction. With kernelSHAP, treeSHAP, and deepKernal subclasses, SHAP can explain any machine learning model's output.

## 3. Results

### 3.1. Statistical Analysis

This study enrolled 198 patients (mild group: 162 cases and severe group: 36 cases), including 80 males and 118 females. The average age of the mild (46.93 ± 14.49 years) and severe (60.97 ± 15.91 years) groups was significantly different. Patients in the mild group were admitted to the hospital 10.40 ± 5.58 days after the onset, which is longer than that in the severe group (8.00 ± 4.88 days, *p* = 0.038). However, the temperature of patients in the severe group was higher than that of those in the mild group (37.42 ± 0.99°C vs. 36.75 ± 0.66°C). Fever, cough, shortness of breath, and dyspnea were significant features associated with the severe group. In terms of basic diseases, 22.22% (8/36) and 6.79% (11/162) of patients in the severe and mild groups, respectively, had high blood pressure (*p* = 0.008) (Table 1).

There were 9.35 ± 7.44 and 6.44 ± 4.08 days between the first CT scan and onset of chest CT features in the mild and the severe groups, respectively. However, the total CT score and the number of different lobes involved in the severe group were significantly higher than those in the mild group. Patients with diffuse (23/36, 63.89%) and large patchy (18/36, 50.00%) appearances were likely to deteriorate. In contrast, patients with diffuse location and patchy shape of the mild group were 35.80% and 81.48%, respectively. Moreover, 80.6% of severe group patients showed lung lesions that had invaded five lobes at admission, compared to 39.5% of the mild patients (*p* = 0.001). The manifestations of pleural effusion, consolidation, crazy paving, and air bronchogram played an essential role in predicting COVID-19 deterioration, indicating that these patients were more likely to develop into severe and critically ill patients (Table 2).

As for laboratory tests, the severe group had a higher WBC count, neutrophil count, and neutrophil ratio and a lower lymphocyte count and lymphocyte ratio than the mild group. Furthermore, the neutrophil-to-lymphocyte ratio (NLR) in the severe group was significantly higher than that in the mild group (8.12 ± 9.69 vs. 3.04 ± 2.75) (Table 1).

### 3.2. Machine Learning Model Performance and Interpretability

A dataset was built, including enrolled patients' clinical information, laboratory tests, and chest CT features. We randomly split the dataset into a 70% training and validation set (138 cases, 113 in the mild group and 25 in the severe group) and a 30% test set (60 cases, 49 in the mild group and 11 in the severe group). Six machine learning models were built, validated, and tested based on the dataset. The performance of the models is reported in Table 3. Five of the six models showed a good fit, except for the DT model with an AUC of 0.707 (95% confidence interval (CI) (0.575, 0.817), *p* = 0.0097). The AUC of XGBoost ranked first for all models, with an AUC of 0.924 (95% CI (0.826, 0.976), *p* < 0.0001). XGBoost achieved 90.91% sensitivity (95% CI (58.7%, 99.8%)) and 97.96% specificity (95% CI (89.10%, 99.90%)). The RF model achieved a 0.907 AUC (95% CI (0.804, 0.967), *p* < 0.0001), 90.91% sensitivity (95% CI (58.7%, 99.8%)), and 95.92% specificity (95% CI (80.4%, 97.7%)). The KNN model obtained a 100% sensitivity (95% CI (71.5%, 100.00%)); however, KNN had a 0.857 AUC (95% CI (0.743, 0.934), *p* < 0.0001) and 61.22% specificity (95% CI (46.2%, 74.8%)). The difference in AUCs between the XGBoost and RF models was not statistically significant (*p* = 0.192). The sensitivity of the two models remained the same; however, XGBoost had higher specificity. Although the AUC between XGBoost and LR, KNN, and RF showed no statistical difference, XGBoost acquired the highest Youden index, sensitivity, and specificity. In general, XGBoost was the best model in this dataset.

**Figure 3:**
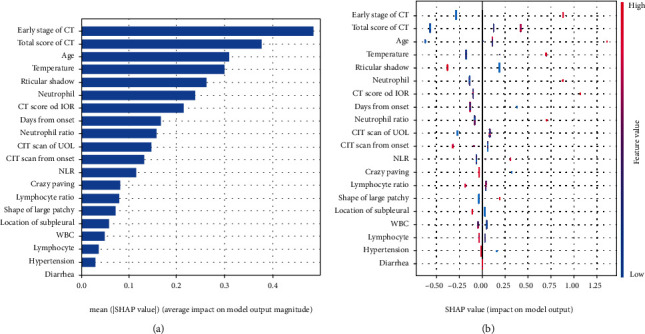
The contribution of various features to the prediction model. The features are listed in descending order according to their contribution to the prediction of a patient becoming severe or critically ill. (a) The importance of features measured by the mean absolute Shapley values according to their contribution. (b) The combination of feature importance and feature effects. The color shows the value of the features from high to low. The horizontal location shows whether the effect of that value caused a higher or lower prediction. Each point is a Shapley value for a feature and an instance.

We further explored the interpretability of XGBoost using the TreeExplainer of SHAP [11].  Figure 3(a) shows the top 19 features that influenced the severe group prediction in descending order. The early stage of chest CT, total CT score of the percentage of lung involvement, and age were the top three contributors to the prediction of deterioration (Figure 3(a)). Patients in the early stage of chest CT at admission were more likely to deteriorate. Moreover, a higher chest CT total score meant that a broader area of the lung was involved; the patients had an increased risk of becoming severe or critically ill (Figure 3(b)). Specifically, injury to the inferior lobe of the right lung (IOR) and upper lobe of the left lung (UOL) had a more significant impact on the prediction than the other lobes.

**Figure 4:**
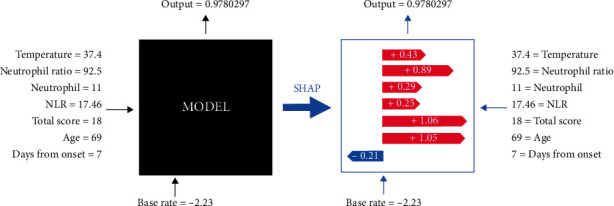
With the help of an interpretable module, we can know how the machine learning model concludes each individual. A 69-year-old patient was predicted to be deteriorating with a possibility of 0.978 (97.8%). The days from symptom onset to hospital admission was seven days, and the temperature at admission was 37.4°C. The neutrophil was 11 × 10^9^/L, with a neutrophil ratio of 92.5% and an NLR of 17.46.

The high neutrophil count, neutrophil ratio, and NLR were also useful in predicting severe and critically ill patients. We can take one step further to explore the feature contribution in individual predictions. The model outputs the probability of a patient becoming severe or critically ill, followed by the specific weight of contribution in the single prediction.  Figure 4 shows an example of a SHAP. While the conventional machine learning model merely outputs the prediction, SHAP was able to show the details of how AI concluded.

## 4. Discussion

The universal manifestation of COVID-19, such as GGO, has low specificity, making it difficult to distinguish COVID-19 from other types of pneumonia solely based on chest CT appearance [12, 13]. It would be even harder, more time-consuming, and often unfeasible for radiologists to assess the disease severity based on the lobar extent, type of pulmonary opacities, clinical information, and laboratory tests, especially in urgent situations or high demand [8, 14, 15]. Since the COVID-19 outbreak, attempts using AI have been made to integrate the information from molecular, medical, and epidemiological scales [16, 17]. The cluster computing power of AI can help with early and improved disease detection and diagnosis, treatment monitoring, and contact tracing of infected individuals, which may help predict the future course of COVID-19 [18]. Moreover, AI can help with designing and developing vaccines and drugs [19–21]. This study took a step further and established six machine learning models to predict COVID-19 patients' prognosis; XGBoost ranked first in performance.

Homayounieh et al. [22] performed multiple logistic regression tests combined with the radiomics of chest CT, clinical information, and laboratory tests on 115 RT-PCR positive patients to predict the possibility of ICU admission, i.e., severe patients. They achieved a 0.84 AUC (95% CI (0.78, 0.85), *p* < 0.02). In comparison, the XGBoost model showed a 0.924 AUC (95% CI (0.826, 0.976), *p* < 0.0001), 90.91% sensitivity (95% CI (58.7%, 99.8%)), and 97.96% specificity (95% CI (89.10%, 99.90%)) based on the clinical information, laboratory tests, and chest CT features. Another issue with AI applications is interpretability. Most AI-predicted models are a “black box”; that is, it is not possible to know further details about each feature's contribution towards model prediction, an important issue with AI applications in clinical settings. Therefore, we established an interpretable XGBoost-based module called SHAP.

This interpretable module outputs the contribution of important features. Patients with features on the list have a higher possibility of deteriorating to severe or critically ill condition. In this cohort, the early stage of chest CT manifestation made the most significant contribution to the prediction, followed by the total score of chest CT and age. Lesions in the severe and critically ill patients seem to be more extensive than mild cases, meaning a higher total score of chest CT and presence of diffused patchy and large patchy appearances on the CT image. Similar to MERS-CoV, patients in the severe group were usually older than those in the mild group, indicating that the elderly tends to develop severe or critical forms of COVID-19, possibly due to comorbidities such as hypertension and underlying immune response [23]. Fever was a typical symptom of COVID-19, and those with a higher temperature at admission were more likely to worsen in the future. The cough was another common symptom, whereas fatigue, shortness of breath, and dyspnea were more common in the severe group, which is consistent with previous research [24, 25]. Furthermore, higher neutrophil count, neutrophil ratio, and NLR ratio increased the possibility of deterioration. Lymphocytopenia is a characteristic of COVID-19 [26]. The virus proliferates in the respiratory system, causing a series of immune responses, leading to changes in lymphocytes and other immune cells [25]. The lower lymphocyte count and lymphocyte ratio, higher WBC and neutrophil counts, and higher neutrophil ratio and NLR may be related to the severity and mortality rate of COVID-19 [27]. Similar to the days from onset to admission, the days from symptom onset to the first CT scan for the severe group were shorter than those for the mild group, meaning that the initial symptoms were serious, resulting in early hospital presentation. In contrast, the lesions appeared to be more extensive in the severe group, suggesting the rapid progression of COVID-19 in these patients. It is worth noting that the more extensive injuries in the IOR and UOL, the more significant their contribution to the deterioration.

With the interpretable machine learning model's application, the medical institutions could identify the potential severe type and critical type patients, hence applying the main observation since admission. Once the crucial factors change during treatment, the doctors could take the early clinical intervention to stop deterioration in the early stage.

Our study has some limitations. First, the small sample size and differences in the number of mild and severe patients may have affected the statistical power of our study. In this study, we applied stratified sampling in data segmentation to reduce the influence brought by imbalanced numbers. Second, the prognostic prediction model may be further improved by combining chest CT radiomics or deep learning models. The application of radiomics and deep learning models may eliminate subjective bias and improve performance. Attempts have been made in a previous study on the detection, outcome, and prognosis prediction of COVID-19 [2, 28, 29]. Third, this was a retrospective study, indicating uncontrollable data loss in the collection, such as procalcitonin and C-reactive protein. In order to ensure a sufficient data size, we had to give up some laboratory results, which may have decreased the performance of the model. Given the limited scale and data, the established XGBoost model requires further clinical validation.

In conclusion, this study established an interpretable machine learning model based on the XGBoost algorithm combined with clinical information, laboratory tests, and chest CT features, aimed at predicting the possibility of COVID-19 patients becoming severe and critically ill, which achieved excellent performance. Furthermore, we explored the most important features in the deterioration process using the interpretable SHAP module, which enabled us to determine the factors that put the patients at risk of developing ARDS and dying from respiratory failure and take necessary clinical interventions to improve the patient prognosis and reduce mortality among the severe and critically ill patients.

## Figures and Tables

**Table 1:** Demographic, clinical characteristics, and laboratory tests of the patients.

	Mild group (*n* = 162)	Severe group (*n* = 36)	*p*
Age (years)			
Mean (SD)	46.93 ± 14.49	60.97 ± 15.91	<0.001
Range	17-81	28-86	
Median age	46	64.50	
Gender			0.513
Male	67 (41.36%)	13 (36.11%)	
Female	95 (58.64%)	23 (63.89%)	
Signs and symptoms at admission			
Days from onset (days)	10.40 ± 5.58	8 ± 4.88	0.038
Temperature (°C)	36.75 ± 0.66	37.42 ± 0.99	<0.001
Fever^∗^	119 (73.46%)	27 (75.00%)	<0.001
Cough^∗^	96 (59.29%)	23 (63.89%)	<0.001
Fatigue	34 (20.99%)	10 (27.78%)	0.352
Shortness of breath^∗^	16 (9.88%)	7 (19.44%)	0.140
Chest tightness^∗^	13 (8.02%)	3 (8.33%)	1
Dyspnea^∗^	6 (3.70%)	7 (19.44%)	0.002
Fear of cold^∗^	6 (3.70%)	5 (13.89%)	0.026
Diarrhea^∗^	8 (4.94%)	1 (2.78%)	1
Headache^∗^	8 (4.94%)	1 (2.78%)	1
Dizziness	4 (2.47%)	3 (8.33%)	0.105
Palpitation^∗^	1 (0.62%)	3 (8.33%)	0.018
Preexisting disease			
Hypertension^∗^	11 (6.79%)	8 (22.22%)	0.008
Diabetes^∗^	6 (3.70%)	4 (11.11%)	0.077
CAD^∗^	5 (3.09%)	2 (5.56%)	0.356
Lung cancer^∗^	0	1 (2.78%)	0.176
Myocardial infarction^∗^	0	1 (2.78%)	0.176
Cerebral infarction^∗^	0	1 (2.78%)	0.176
Tuberculosis^∗^	0	1 (2.78%)	0.176
Laboratory tests			
WBC (×10^9^/L)	5.53 ± 2.30	7.11 ± 3.53	0.014
Neutrophil (×10^9^/L)	3.61 ± 2.10	5.80 ± 3.50	0.001
Neutrophil ratio (%)	62.48 ± 13.15	76.15 ± 12.11	<0.001
Lymphocyte (×10^9^/L)	1.40 ± 0.50	0.99 ± 0.47	<0.001
Lymphocyte ratio (%)	27.33 ± 10.07	16.80 ± 9.71	<0.001
NLR	3.04 ± 2.75	8.12 ± 9.69	0.004

CAD: coronary artery disease; WBC: white blood cell; NLR: neutrophil-to-lymphocyte ratio. ^∗^Fisher's exact test.

**Table 2:** Chest CT features of the patients.

	Mild group (*n* = 162)	Severe group (*n* = 36)	*p*
Stage			0.208
Early stage	44 (27.16%)	10 (27.78%)	
Progress stage	105 (64.81%)	26 (72.22%)	
Restoration stage	13 (8.02%)	0	
Location			0.002
Subpleural	50 (30.86%)	2 (5.56%)	
Scatter	54 (33.33%)	11 (30.56%)	
Diffuse	58 (35.80%)	23 (63.89%)	
Shape			<0.001
Nodular	11 (6.79%)	1 (2.78%)	
Patchy	132 (81.48%)	17 (47.22%)	
Large patchy	19 (11.73%)	18 (50.00%)	
Number of lobes involved			<0.001
1	22 (13.58%)	0	
2	22 (13.58%)	1 (2.78%)	
3	20 (12.35%)	1 (2.78%)	
4	34 (20.99%)	5 (13.89%)	
5	64 (39.51%)	29 (80.56%)	
Image manifestations			
Pleural effusion	1 (0.62%)	4 (11.11%)	0.004
Fibrosis	64 (39.51%)	15 (41.67%)	0.811
Consolidation	85 (52.47%)	28 (77.78%)	0.006
Reticular shadow	95 (58.64%)	34 (94.44%)	<0.001
Crazy paving	9 (5.56%)	15 (41.67%)	<0.001
Air bronchogram	55 (33.95%)	26 (72.22%)	<0.001
Pleural thickening	62 (38.27%)	24 (66.67%)	0.002
Lymphadenovarix	10 (6.17%)	4 (11.11%)	0.493
GGO	162 (100.00%)	36 (100.00%)	—
Nodules	68 (41.98%)	19 (52.78%)	0.211
Quantitative features			
CT from onset (days)	9.36 ± 7.44	6.44 ± 4.08	0.002
Total score	4.24 ± 2.54	8.50 ± 4.44	<0.001
UOR	0.75 ± 0.65	1.75 ± 1.23	<0.001
MOR	0.62 ± 0.66	1.36 ± 0.90	<0.001
IOR	1.10 ± 0.68	2.00 ± 1.20	<0.001
UOL	0.73 ± 0.59	1.53 ± 0.97	<0.001
IOL	1.04 ± 0.67	1.86 ± 1.13	<0.001

GGO: ground-glass opacity; UOR: upper lobe of right lung; MOR: middle lobe of right lung; IOR: inferior lobe of right lung; UOL: upper lobe of left lung; IOL: inferior lobe of left lung.

**Table 3:** The AUC, sensitivity, and specificity comparisons.

	AUC (95% CI)	Sensitivity (95% CI)	Specificity (95% CI)	*p*
LR	0.891 (0.783, 0.956)	90.91 (58.7, 99.8)	93.88 (83.1, 98.7)	0.1306
KNN	0.857 (0.743, 0.934)	100.00 (71.5, 100.0)	61.22 (46.2, 74.8)	0.2844
DT	0.707 (0.575, 0.817)	45.45 (16.7, 76.6)	95.92 (86.0, 99.5)	0.0095
RF	0.907 (0.804, 0.967)	90.91 (58.7, 99.8)	95.92 (86.0, 99.5)	0.1915
SVM	0.892 (0.785, 0.958)	90.91 (58.7, 99.8)	91.84 (80.4, 97.7)	0.2006
XGBoost	0.924 (0.826, 0.976)	90.91 (58.7, 99.8)	97.96 (89.1, 99.9)	—

Two-sided *p* values were calculated by comparing AUC for the XGBoost model with the other models. AUC comparisons were evaluated using the DeLong test; LR: logistic regression; KNN: *k*-nearest neighbor; DT: decision tree; RF: random forest; SVM: support vector machine; XGBoost: eXtreme gradient boosting.

## Data Availability

All data used to support the findings of this study are restricted by the Ethics Committee of Honghu People's Hospital in order to protect patient privacy.
